# The Pay of the Nurse: II. Under-paid Private Nurses

**Published:** 1921-03-12

**Authors:** 


### THE MATRONS' AND SISTERS' DEPARTMENT.

### THE PAY OF THE NURSE.

### II. Under-paid Private Nurses.

In our last issue we examined into the value of the trained district nurse as compared with that of the totally untrained girl of nineteen, and we found that, so far from having benefited from an economic point of view by the years of training she had received, she was actually in a worse position than before she entered her hospital.

We have now to examine into the position of the woman who embraces the career of private nursing. What is her value when she has completed her training and practises her profession? We have been at some pains to examine the advertisements for private nurses in one of the best-known nursing papers, because advertisements at any rate show which way the wind is blowing. People do not put a fancy value on the services of employees whose services they desire to secure, nor do they fruitlessly advertise vacant posts at a salary no one will accept. We find that the market value of a totally untrained "attendant" is estimated at £71 a year, with board, lodging, laundry, and uniform for a forty-eight-hour week. We find that "assistant nurses, Class II.," without previous experience, can command £50, with full emoluments for a fifty-hour week. And we turn to see what the woman who has had a three-year hospital course and passed various examinations is offered in precisely the same profession and without any limit to the hours of work. It is not encouraging. A considerable number of nursing institutes advertise for fully-trained nurses. Sometimes they specify theatre or good surgical qualifications. Sometimes they want fever qualifications in addition. And the rate of pay they offer is pretty steadily £60 to £65 per annum, without allowance for uniform or laundry. In one case we have £60 to £70 offered, with the proviso that the applicants must show certificates from "a well-known training-school"; not, be it observed, a hospital containing a certain number of beds, but a training-school with a *cachet*; the nurse must be armed with an air of fashion if she is to be worth her £60 a year. In some cases £50 is offered, with a 5 per cent. commission on earnings. In others there is the promise of an extra £5 a year for the C.M.B. or other additional certificate, a curious comment on the circumstance that each such certificate takes at least six months to earn and often costs over £30 in solid fees. For a nursing home the salary offered the trained nurse is £50 to £55.

The rate of pay which these advertisements disclose is beyond measure deplorable. We are not disposed to blame the authorities who offer £71 to untrained attendants. They would no doubt pay less if they could secure the women they want for less. But what we do insist is that nurses have got caught in a vicious circle, round which they revolve in complete inability to find their way out. While we have been discussing the question whether or no their weekly fee should be three guineas or three guineas and a-half, it begins to appear that their total earnings, when upon a salaried basis, are about £55 to £70 a year, which is about what they were worth untrained in other branches of the same profession.

In the last number of the College *Bulletin* appears a letter from a private nurse which is worth attention. She shows that few nurses on the co-operative system can count on more than thirty-five weeks' work in the year, and that when the necessary deductions have been made for board during the idle season and for the upkeep of uniform the net profits amount to no more than £55 a year, even when the weekly fee is £3 3s. a week. It would be extremely interesting to have a few more budgets of this nature. It would bring to the notice of the public and the doctors who employ nurses the fact that the profits of trained nurses, even at the top of their profession, are far less than is usually supposed, and that the dearth of probationers for this underpaid profession is no more than the outcome of a protective instinct.

If hospital training is so valuable as we know it to be, why does it not raise the market value of those who surrender so much time to secure it? We glance at the advertisements for manual workers. We find that a nursery nurse can command £60 a year; that cooks are eagerly advertised for at £50 and £55, with uniform; that a laundress can command £54 and £55; that even a kitchen-maid and a matron's maid can get £40. Why, then, does any one expect to get "trained nurses with C.M.B." for private nursing at £52 a year? The world is out of joint indeed, and private nurses are being made to feel the full weight of other folk's poverty. We turn to advertisements for hospital maids, in which a salary of 30s. a week is offered, plus 20s. a week war bonus. Deducting a charge for meals, this works out at about £70 a year, with uniform and a prospect of a pension, for ninety-six hours' work a fortnight. If money were all, a group of trained nurses could run the domestic side of the establishment *en famille*, and considerably better their financial prospects by the change.

*(To be continued.)*

